# Intrafat Sequestration of Artemisinin Disguised as a Purulent Collection during a Posterolateral Hip Approach

**DOI:** 10.1155/2019/6984875

**Published:** 2019-05-21

**Authors:** Atchi Walla, Batomayena Bakoma, Pilakimwé Egbohou

**Affiliations:** ^1^Department of Orthopaedics, Campus Medical Teaching Hospital, Lomé, Togo; ^2^Medical School, University of Lomé, Togo; ^3^Department of Anesthesia, Sylvanus Olympio Teaching Hospital, Lomé, Togo

## Abstract

The posterolateral hip approach is the oldest and most used way to implant total hip arthroplasty. The anterior part of the oblique portion of this posterolateral approach corresponds more or less to the superolateral quadrant of the buttock in which the intramuscular injection of various drugs, including the compounds derived from artemisinin, is carried out. Thus, in a malarial endemic area where gluteal injections of the compounds derived from artemisinin are not rare, poor performance of an injection by the deposition of the product in the fat and not deeply in the muscle can be at the origin of the sequestration of the drug in adipose tissue and give the macroscopic appearance of a pus. The authors present a case of intrafat sequestration of artemisinin taken for purulent collection during a posterolateral hip approach for total hip arthroplasty.

## 1. Introduction

The posterolateral approach of hip is the oldest and most used way to implant total hip arthroplasty (THA) [1]. The posterior part of this approach is located on the line joining the posterior inferior iliac spine and the summit of the greater trochanter [2]. In this portion, before reaching the fascia of the gluteus maximus muscle after the skin incision, one passes through an adipose layer which is all thicker as the patient is obese and female.

The anterior part of this oblique portion of the posterolateral approach corresponds more or less to the superolateral quadrant of the buttock in which the injection of various intramuscular drugs such as artemisinin-derived compounds is done.

Thus, in a malaria-endemic area where gluteal injection of artemisinin is not uncommon [3], poor injection performance by depositing the product in the fat and not deep in the muscle may be the cause of its sequestration in adipose tissue and give the macroscopic appearance of a pus. It can be a potential pitfall arising in a common surgical gluteal approach for THA.

The authors present a case of intrafat sequestration of artemisinin taken for purulent collection during a posterolateral hip approach for total arthroplasty. This case seems to be the first of its kind.

## 2. Case Presentation

The patient was a 65-year-old woman without notable antecedents presented to our institution for progressive left hip pain for approximately 8 months. It was a mechanical pain of the hip well relieved by the usual analgesics. The appearance of walking distance and the poor response to analgesics forced her to consult in our center.

**Figure 1:**
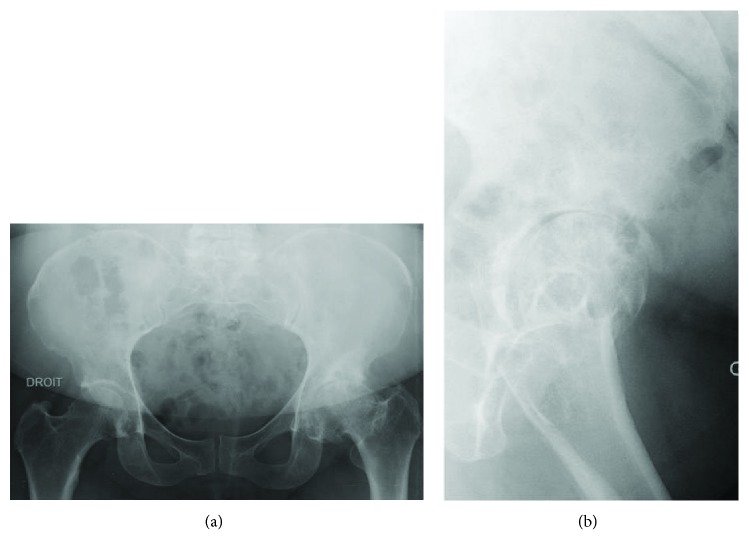
(a) Pelvic AP radiograph with left hip osteoarthritis. (b) Lateral left hip radiograph with osteoarthritis.

The BMI was 35,5. The walk was almost normal. There was no cutaneous scar on the lateral side of the left hip or on the ipsilateral buttock. There was a good trophicity of the abductors. Lateral rotation and abduction were markedly diminished. The rest of the exam was strictly normal. The pelvis AP (Figure 1(a)) and lateral (Figure 1(b)) left hip radiographs revealed signs of hip osteoarthritis. We concluded that it was a symptomatic left hip osteoarthritis that was more and more disabling in an obese woman of 71 years with no particular history. We indicated THA by posterolateral approach.

**Figure 2:**
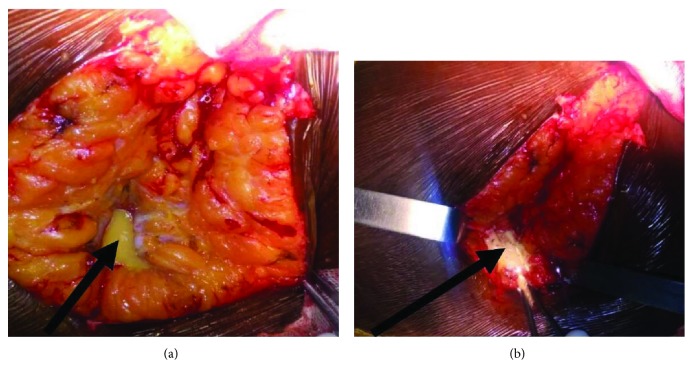
(a) Left hip posterolateral approach. Note the yellowish-white, thick, and not really oily aspect of the collection. (b) Evocative appearance of a purulent collection.

In the operating room, after the skin incision and subcutaneous haemostasis, we discovered in the adipose tissue about 5 cm thick a kind of well-circumscribed shell of about 2.5 cm of axis. Her incision gave rise to a whitish, thick color, looks a little oily collection (Figure 2(a)), resembling a purulent collection (Figure 2(b)). A sample for bacteriological investigation in a lab was carried out. The hull with its clear boundaries within the gluteal fat was resected and entrusted to the pathologist. All the neighborhood tissues were healthy (very localized lesion).

In front of this collection which appeared to be purulent, we limited ourselves to the resection of this hull, the cleaning of the wound, and the deferred implantation of the prosthesis.

Cytobacteriological examination of the specimen revealed its greasy appearance, epithelial and lymphocytic cells; there were no visible germs. Histological examination of the resected shell revealed a fibrous wall with chronic inflammatory remodeling made of lymphocytes and plasma cells with no necrosis centers.

In the light of these laboratory results, we conducted the interview of the patient, who reported a notion of malaria for about two months to the screen treated with an intramuscular injection on the right buttock of the compounds derived from artemisinin. We found the result of the thick drop before the injection which was positive and that of the injection which had not been negated; the patient was then successfully treated orally. The sample was sent to a lab for confirmation by artemether identification by thin layer chromatography (TLC).

A sample of 40 g of human fat was treated with ethyl acetate (50 ml × 3) after filtration on Whatman paper, the solvent was evaporated, and the residue was taken up with acetone (40 ml) constituting the sample to be analyzed. Artemether was purchased from a local pharmaceutical company.

Implementation of the TLC: solution to be analyzed: 20 *μ*l of sample; control: artemether (80 mg/mL), 10 *μ*l deposit; support: silica gel GF254; mobile phase (10 ml): dichloromethane, ethyl acetate (7/3); and developer: 25 ml anisaldehyde reagent, 5 ml concentrated acetic acid, 450 ml ethanol, and 25 ml concentrated sulfuric acid. Using a capillary tube, 20 *μ*l of the sample was deposited on the plate (silica gel GF254), the control 10 *μ*l. The plate is placed in a tank previously saturated with the migration or elution solvent (mobile phase) which covers the bottom of the tank at 5 mm height. The migration of eluting solvent causes the substances contained in the samples at various speeds; spots are formed characterizing the substances present in the sample.

The plate was removed from the tank as soon as the solvent front reached about 9 cm. The plate was dried and observed under a UV lamp at 254 nm and then revealed with the developer which will characterize the artemether in human fat.

**Figure 3:**
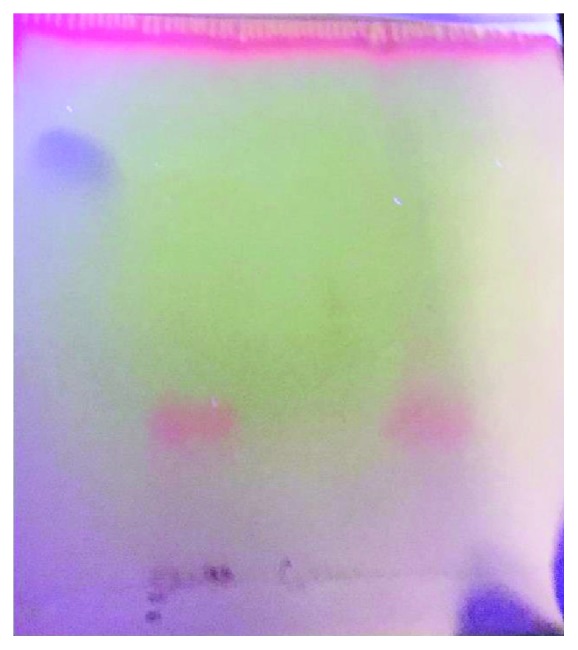
TLC plate for artemether identification. Note the orange spot on the left side of the sample and a spot with the same color on the right side of the control. The two spots have the same front report.

The plate then shows an orange spot on the left side of the sample and a spot with the same color on the right side of the control; the two spots have the same front report as shown in Figure 3. This indicates that there was artemether in this human fat sample.

## 3. Discussion

THA is considered as one of the most successful surgical procedures providing pain relief and function in patients with end-stage hip osteoarthritis [4–6]. More than one million THA are being performed worldwide towards the world, and this figure could double over the next two decades [7].

Whether classic Moore or miniinvasive, the posterolateral approach of the hip remains for many orthopedists today, the surgical means of access to the hip for prosthetic surgery [1].

This posterolateral approach crosses the gluteal region which is anatomically rich. This region has an important subcutaneous fat plan that is physiologically greasy.

On this approach, there may be traps even in the patient prepared for prosthetic hip surgery, such traps include occult fat tumors which are asymptomatic subcutaneous tissue lipomas recognizable by their small size; they are rarely encountered in adults; macroscopically, it is difficult to differentiate it from normal fat [8, 9].

It may also be in this region, pyomyositis which are deeper primitive bacterial infections of the gluteal region; in this case, the clinical context marked by the signs of infection and the rich biology allowed easy diagnosis. Again, it is an affection of the child and adolescent [10, 11].

This case, we report which probably is the first one, indicates that it is possible to be in the gluteal region, in front of an intrafat sequestration of artemisinin, in particular in the patients resident or coming from an endemic area where the gluteal intramuscular injections of the compounds derived from artemisinin are particularly common; this is all the more likely that the patient is female with a high BMI.

The history of more or less recent injection of the compounds derived from artemisinin into the buttock, the absence of clinical and biological signs of infection, and the painless oily appearance of the collection should point towards artemisinin sequestration. The search for a more or less recent concept of malaria and the mode of its treatment in this type of patient becomes important to specify.

Furthermore, in countries where malaria is endemic, sensitization of health care workers on the use of artemisinin by injection only recommended in severe malaria must be intensify. In the case of severe malaria where artemisinin injection is indicated, workers have to use an appropriate needle in at least one overweight patient in order to avoid the untimely filing of drug in the fatty layer of the gluteal region in overweight or fat patients.

## 4. Conclusion

It is necessary to have in mind the intrafat sequestration of the compounds derived from artemisinin during the posterolateral hip approach in patients resident or coming from a malaria-endemic area with a high BMI and not to be confused with a purulent collection.

Continuing education of health care providers for good practices of intramuscular injection of the compounds derived from artemisinin by the use of adequate length of needles in at least overweight people was done.
